# Designing an mHealth App for Stroke Rehabilitation in Indonesia: Mixed Methods Design Science Research Study

**DOI:** 10.2196/91464

**Published:** 2026-07-23

**Authors:** Sabrina Atha Shania, Putu Wuri Handayani, Kamila Alifia Imanuddin

**Affiliations:** 1 Faculty of Computer Science, University of Indonesia, Jl Kampus UI, Depok, West Java, 16424, Indonesia, 62 81380883402

**Keywords:** stroke, rehabilitation, mobile health, mHealth, design science research, intervention theory, Indonesia

## Abstract

**Background:**

Indonesia has the second-highest prevalence of stroke in Southeast Asia, with 4,918,487 people (1.7% of the population) living with this condition as of 2025. This is attributable to the living conditions in Indonesia, where limited health care workers and facilities are available to assist with stroke rehabilitation via mobile health (mHealth) interventions.

**Objective:**

This study designed an mHealth app to facilitate stroke rehabilitation in Indonesia.

**Methods:**

In this mixed methods study, interviews were conducted with 10 experts (doctors, therapists, and medical students) and 8 patients who have experienced a stroke to explore the need for stroke rehabilitation management apps. Using a design science research approach, we conducted 3 iterative design cycles, each comprising design, development, and evaluation, to construct an mHealth app for stroke rehabilitation. The first iteration produced a low-fidelity app prototype informed by intervention theory. The prototype, which comprised 13 features for patients and 12 for medical personnel, was evaluated through interviews with medical personnel. The second iteration produced a design for a high-fidelity app prototype based on feedback from the first iteration. The third iteration yielded an improved app based on feedback from the second iteration.

**Results:**

This study identified app features that support patient rehabilitation, including patient training programs with progress monitoring in functional, speech, and occupational therapy, along with consultations and home visits to connect rehabilitating patients with medical personnel. Other features of the app include onboarding, gamification, users’ authentication, user profile management, schedule reminders, activity targets, therapeutic progress reports, and educational materials and videos. The prototypes received a System Usability Scale score of 71.83 (“good”) and average scores of 2.32 (system usefulness), 2.39 (information quality), 2.42 (interface quality), and 2.35 (overall).

**Conclusions:**

This study provides guidance for health care facilities and regulators to improve the quality of rehabilitation services for patients with stroke in Indonesia.

## Introduction

### Background

Stroke can cause a wide range of long-term symptoms and remains a significant global health burden, requiring comprehensive and innovative rehabilitative approaches to optimize recovery outcomes [1]. The World Health Organization (WHO) [2] reports that every patient with acute stroke or cognitive dysfunction should undergo an initial functional assessment to determine rehabilitation needs and develop an individualized rehabilitation plan. The Barrow Neurological Institute [3] argues that most patients with stroke (80%‐90%) require some form of rehabilitation, whether acute, subacute, inpatient, or outpatient.

Various types of rehabilitation are available for patients with stroke, including occupational therapy, speech therapy, and physiotherapy [4]. Occupational therapists teach patients with stroke how to carry out daily living activities, such as cooking, cleaning the house, and taking care of themselves [4]. Speech therapists draw on scientific and technological knowledge to help patients communicate (touching on speech, voice, fluency, and other aspects of language) and cope with swallowing disorders [5]. However, the implementation of stroke rehabilitation faces significant and persistent challenges, including patient noncompliance and the gap between the need for post–inpatient intensive therapy and its actual availability in health care systems [6]. Globally, the cost of treating stroke in the year 2022 was estimated to reach more than US $721 billion, or about 0.66% of the world’s gross domestic product [7].

Indonesia has the second-highest incidence and prevalence of stroke in Southeast Asia, with 642,943 new cases recorded and 4,918,487 people living with stroke between 2017 and 2022 [8]. In 2023, stroke ranked third among catastrophic diseases in Indonesia [9]. Stroke prevalence differs by age group, with individuals aged 75 years and older demonstrating the highest prevalence (41.3 per 1000 people) and the 15 to 54 years age group exhibiting the lowest at less than 9 per 1000 [10].

The challenges of stroke rehabilitation in developing countries, such as Indonesia, include health care factors, such as the demands of a growing health system and a lack of income for specialized training, rehabilitation facilities, and rehabilitation staff [11]. Other challenges include a lack of adequate learning opportunities and formal education programs for nurses, therapists, and even doctors [12]. There are also internal challenges on the patient side. Kayola et al [11] note that individual-level factors, such as low health literacy, can increase drug and rehabilitation costs as well as caregiver wages. Poststroke traumatic stress disorder can lead to nonadherence to treatment, increase disability rates, and increase the frequency of lesions in the right brain and brainstem [13].

Mobile health (mHealth) can provide innovative solutions to improve rehabilitation efficiency, offer more personalized services, and help health care facilities better understand patient needs [14-16]. Szeto et al [17] explain that the most beneficial apps integrate therapeutic elements such as social interaction, real-time feedback, rhythmic cues, and multisensory stimulation, as well as repetitive, task-specific, and/or goal-oriented exercises. However, user compliance with rehabilitation remains relatively low, and existing apps lack interoperability with electronic health records [17]. Song et al [18] discuss apps that specify daily rehabilitation exercises via asynchronous video recordings monitored by clinicians and offer reminder features to boost patient adherence. However, most of these studies were conducted in developed countries, including Spain, the Netherlands, the United States, and the United Kingdom [15].

Design science research (DSR) is a methodology that seeks to overcome real-world challenges through the design and evaluation of innovative artifacts [19]. DSR was used as this study’s main methodological framework to ensure that the artifact, in this study named as a prototype, would be developed appropriately for users in Indonesia. Additionally, intervention theory is used as a foundation for designing more effective problem-solving systems [20]. Intervention theory holds that user behavior can be influenced through 3 main principles: the provision of valid and useful information, the freedom of the user to act based on the information obtained, and the formation of an internal commitment to the chosen action [20].

### Objective

This research aimed to design an mHealth app for stroke rehabilitation management, focusing on the promotive, preventive, curative, and rehabilitative aspects. Our research question was as follows: How can an mHealth app for stroke rehabilitation in Indonesia be designed based on DSR and intervention theory? This research provides guidance for the development of health apps that meet the needs and expectations of patients with stroke in Indonesia.

## Methods

### Ethical Considerations

This research was carried out in collaboration with Indonesia’s National Brain Center Hospital and received ethical approval from Mahar Mardjono on September 26, 2025 (reference DP.04.03/D.XXIII.9/208/2025). All respondents provided written informed consent to participate, and the data submitted were anonymous. The respondent data were used only for the purposes of this research.

### Study Design

This mixed methods study used both quantitative and qualitative approaches. This research followed the 5 stages of the DSR methodology: problem identification and motivation, definition of solution objectives, design and development, demonstration, and evaluation and communication [21]. We collected data at each stage through interviews. However, for the evaluation and communication stage, we used both interviews (qualitative approach) and questionnaires (quantitative approach) to evaluate the prototypes. We triangulated the results to ensure that the prototypes aligned with the respondents’ needs.

Using design science research, we conducted 3 iterative design cycles, each comprising design, development, and evaluation, to construct an mHealth app for stroke rehabilitation. The first iteration—the problem identification and motivation stage—was carried out through a literature review, application benchmarking, and interviews with patients with stroke and experts (including doctors, therapists, and medical students), who provided written consent. A thematic analysis [22] of the interview data was conducted to group the speakers’ statements into problem categories. These categories allow for the simplification of open-ended data and indicate the problems’ frequencies and levels of validation. Based on these findings, we proceeded to define the objectives and establish what would qualify as solutions, following the “how might we” (HMW) approach [23]. Mapping with the HMW approach offered solutions and ideas for features of the proposed app. To ensure the relevance of the app to user needs, we defined personas. Personas represented concrete pictures of user characteristics [24], making the entire mHealth solution design process more targeted and contextual. The next stage was design and development; we aimed to develop solutions in the form of low-fidelity prototypes, using the principle of the 8 golden rules as the interface design principle [21]. The last stage of the first iteration was demonstration, as we tested the prototype through interviews with doctors, therapists, and health students. Each stage of the first iteration included an evaluation and validation mechanism to ensure that the developed artifact matched the users’ needs.

The second iteration focused on refining the prototype design based on the evaluation results from the first phase. The second iteration returned to design and development, using input in the form of feedback on the previous low-fidelity prototype [21]. This feedback guided the design of a higher-fidelity prototype in accordance with the 8 golden rules [21]. After the artifacts were repaired and determined suitable for our purposes, we proceeded to evaluation and communication [21]. This stage included usability testing via the System Usability Scale (SUS), as well as interviews with patients and experts (doctors, therapists, and medical students). This evaluation aimed to explore user perceptions and experiences and to map obstacles and potential improvements to the interface and features [25]. This iteration yielded more targeted design recommendations and laid the foundation for the third phase.

**Figure 1.**
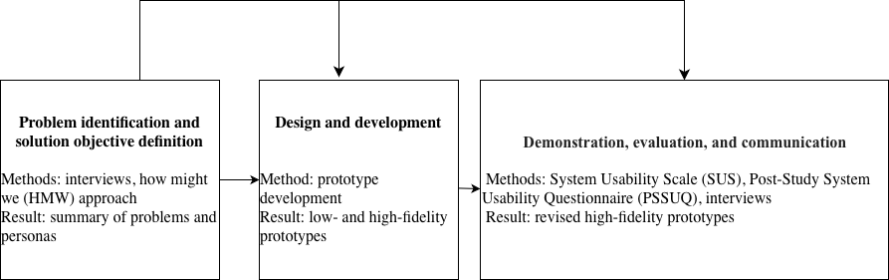
Research flow based on design science research.

The third and final iteration of the DSR development process began with design and development based on feedback and recommendations from the second stage. We then returned to evaluation and communication by measuring the satisfaction and effectiveness of the system [21]. For this, we used the Post-Study System Usability Questionnaire (PSSUQ) with various respondents. After ensuring that the evaluation results met the criteria, we proceeded to formulate design principles. This involved cross-iteration analysis to develop conceptual design principles based on user needs, feature responses, and solution effectiveness. SUS and PSSUQ have been widely adopted to assess the usability of digital systems [25]. The final stage was the preparation of conclusions and recommendations that summarized all findings [21]. Figure 1 describes the research flow based on DSR.

### Inclusion Criteria

We recruited patients who have experienced a stroke, experts in stroke rehabilitation, and respondents interested in stroke rehabilitation to obtain in-depth data. Patients treated at the National Brain Center Hospital were selected if they were 18 to 80 years old, had experienced a stroke, and experienced cognitive or physical impairments. Experts were selected if they were medical rehabilitation specialists, therapists, or students with a health-related major who were willing to participate (purposive sampling). Table 1 describes the respondent demographics for each DSR stage.

**Table 1.** Demographics of respondents.

DSR^a^ stage, data collection method, and respondents’ role	Respondents, n
Problem and solution objective identification	
Interview	
Experts	10
Patients with stroke	8
Design and development	
Prototype development	
No respondents involved	0
Demonstration, evaluation, and communication	
SUS^b^ questionnaire	
Patients with stroke	14
Caregivers of patients with stroke	109
Health care workers	23
PSSUQ^c^ questionnaire	
Health care workers	14
Patients with stroke	41
Caregivers of patients with stroke	73
Interview	
Health care workers	20
Patients with stroke	5

a DSR: design science research.

b SUS: System Usability Scale.

c PSSUQ: Post-Study System Usability Questionnaire.

### Analysis Methods

The data were analyzed using 2 approaches. Qualitatively, we identified important characteristics or features required in the app design; quantitatively, we evaluated and measured the quality of the app prototypes. The analysis process began in the first iteration with semistructured interviews to identify user needs. In the next stage, we implemented a thematic analysis technique proposed by Ozuem et al [22]. This involved classifying and grouping problems while counting the number of respondents who confirmed each problem. Once categorized, the problems were mapped onto questions. The HMW framework was used as a guide to develop answers to each question [23]. We then converted the data into persona profiles that described the users’ characteristics. The information architecture design was also developed at this stage to systematically organize the app content and facilitate user accessibility according to the corresponding personas. Semistructured interviews were conducted as a form of design evaluation in the second and third iterations. The evaluation was carried out using 2 evaluation tools—SUS and PSSUQ—to analyze 3 key components: system usability, information quality, and interface quality [25].

### Research Instruments

Questionnaires and interviews were used during the evaluation stage. The questionnaire was conducted in the second and third iterations. In the second iteration, the questionnaire consisted of questions about demographics and the use of health apps. This section addressed patients who experienced stroke. The demographic questions touched on age, residence, occupation, number of devices, and email. Questions about the use of health apps covered experiences with mHealth related to stroke rehabilitation (such as app names and disadvantages). In the third iteration, results from the SUS questionnaire in the second iteration and the PSSUQ in the third iteration were used to test usability levels and questions about stroke rehabilitation. The SUS uses a 5-point Likert scale, where 1 indicates “strong disagreement” and 5 indicates “strong agreement,” while the PSSUQ uses a different Likert scale, where 7 indicates “strong disagreement” and 1 indicates “strong agreement.” Multimedia Appendix 1 further explains SUS, and Multimedia Appendix 2 details PSSUQ. Multimedia Appendix 3 presents the interview questions used for each respondent.

## Results

### Identifying Problems and Defining Solutions

This stage began with a literature review on the epidemiology of stroke globally and in Indonesia, as well as challenges in handling stroke rehabilitation. We also conducted interviews with 10 health workers (general practitioners, medical students, and therapists) and 8 patients who have experienced a stroke (or their caregivers as intermediaries) to understand patients’ needs and challenges. Multimedia Appendix 4 summarizes the problems emerging from interviews with 18 respondents, comprising 10 experts and 8 patients. These respondents identified various problems in stroke rehabilitation, including a lack of self-rehabilitation exercises at home, low patient compliance with exercises, unclear roles and low willingness among medical personnel, the decreased ability of patients to undergo rehabilitation in the hospital due to age, limited health care facilities, the variety of conditions among patients who have experienced a stroke, and the absence of evidence on home exercises as a form of evaluation, which leaves evaluations reliant solely on patient reports, which are often subjective and not always accurate.

Problem solution mapping was used to understand these problems and find solutions. This study adopted the HMW method, in which questions were formulated based on identified problems. The research continued by exploring all possible solutions that were relevant and effective for solving the problems. The solutions discovered through the HMW method are summarized in Multimedia Appendix 5.

Subsequently, personas were constructed to understand users’ needs, motivations, and goals in stroke rehabilitation. Based on the previous stages, 2 main personas with different characteristics were identified: health care workers and patients who experienced a stroke. These 2 types have different roles and needs in stroke rehabilitation management. Thus, the app was designed for these individuals.

The patient persona represents a man who had a stroke at the age of 56 years and worked as a lawyer. Currently, he is undergoing a physical rehabilitation process at home with the support of his family members. The stroke he experienced impacted his physical abilities, especially in terms of mobility and body coordination. In addition, he experiences communication disorders, which make his pronunciation unclear. Such conditions make it hard for patients to remember the frequency and types of exercise that need to be performed after discharge from the hospital, while also making it difficult to visit the hospital due to cognitive impairment. This persona has 2 basic needs in his rehabilitation process: the support and encouragement of his family or friends to maintain his spirit, and the presence of his family to help guide him through the rehabilitation process.

The persona of the medical personnel represents a female who is aged 23 years and lives in Jakarta. As a professional in physical rehabilitation, she has 2 years of experience as a physiotherapist. She has treated several patients who have experienced a stroke, but the rehabilitation process has not always proceeded smoothly. She recognizes the challenges in dealing with patients who have experienced a stroke, which involve both internal patient factors and external factors that affect the recovery process. She has 2 basic needs in carrying out her duties as a physiotherapist: the ability to effectively manage post-stroke rehabilitation, and the need to improve both her nonclinical and clinical skills to offer optimal care. Because she is motivated, she has a realistic perspective and always appreciates patients’ progress. However, she has difficulty motivating patients to undergo stroke rehabilitation consistently and is unable to monitor patients’ exercises at home.

### Design and Development

**Figure 2.**
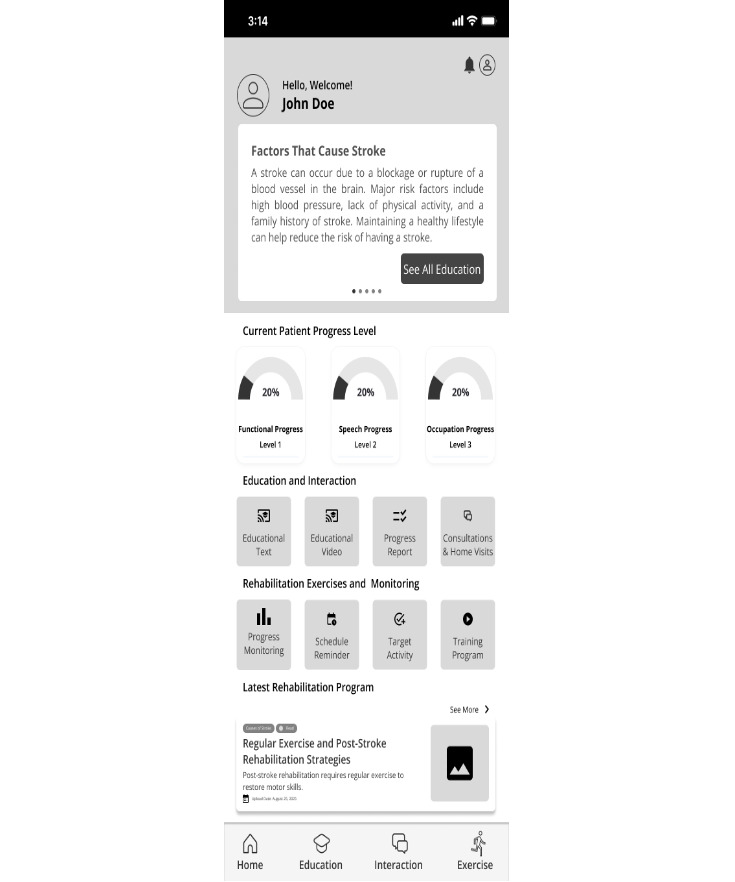
Low-fidelity home screen.

**Figure 3.**
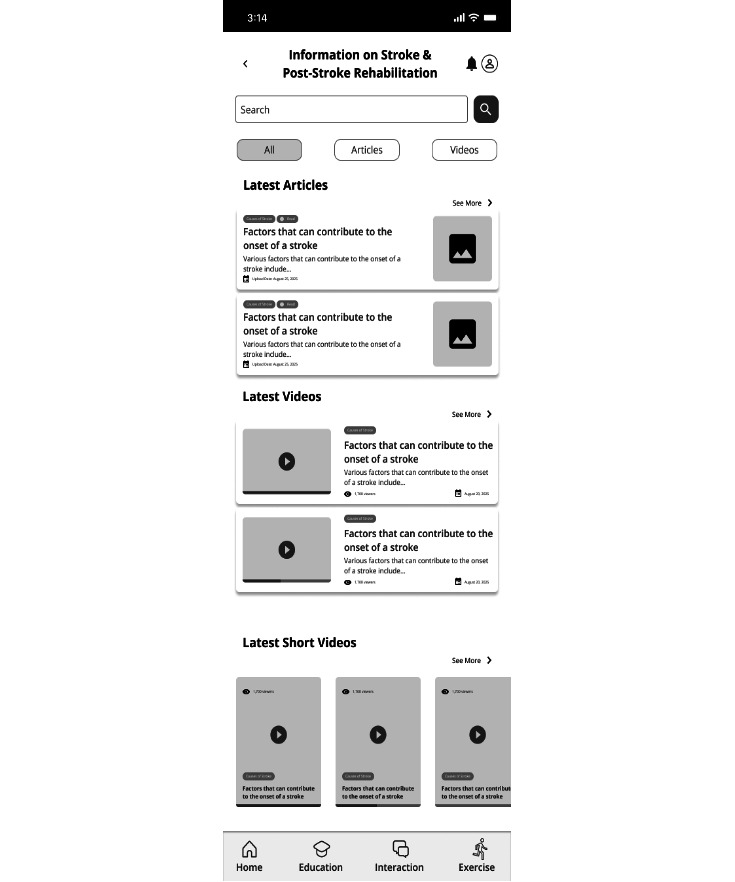
Low-fidelity stroke education.

**Figure 4.**
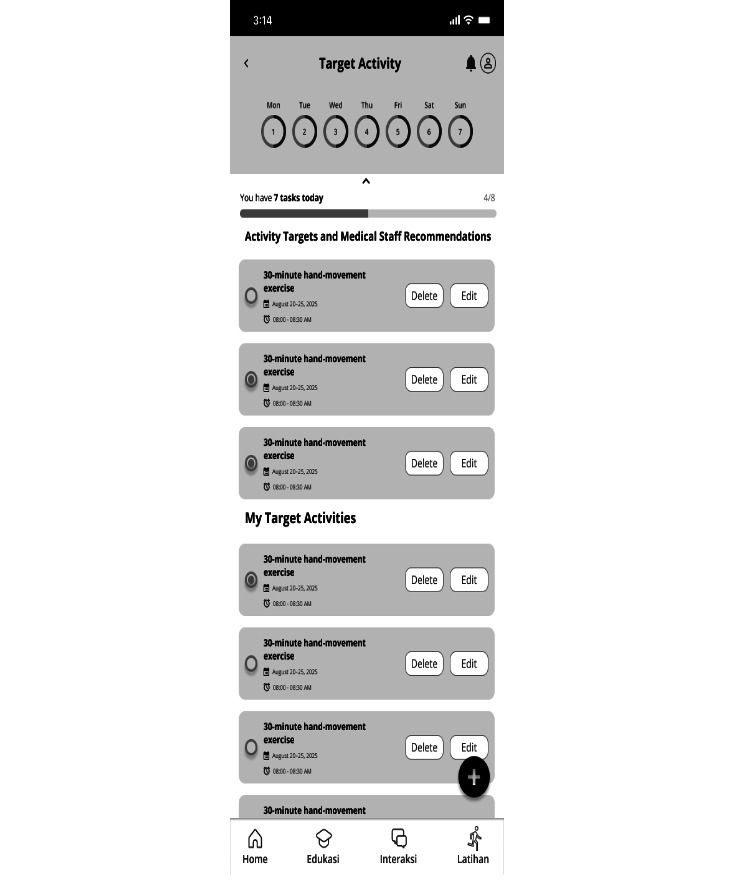
Low-fidelity stroke activity.

**Figure 5.**
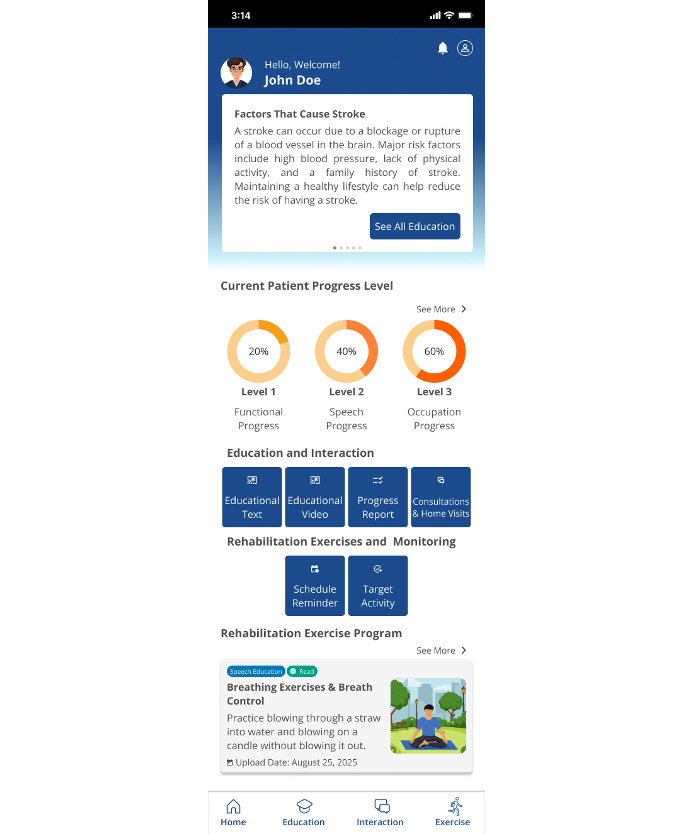
High-fidelity home screen.

**Figure 6.**
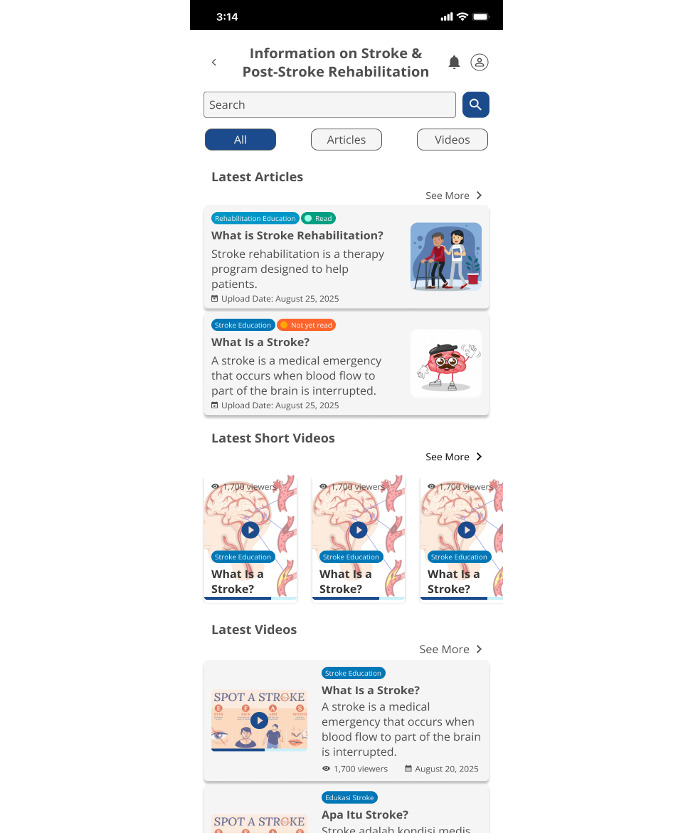
High-fidelity stroke education.

**Figure 7.**
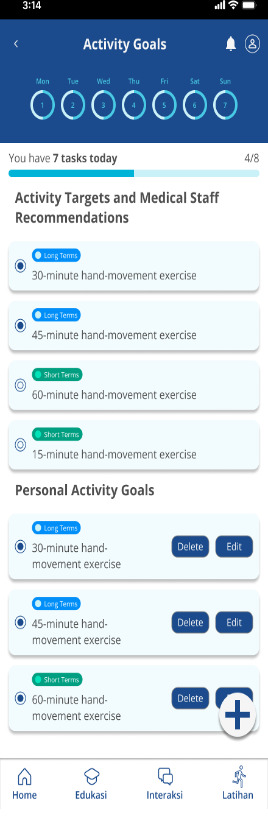
High-fidelity stroke activity.

Based on the mapping of problems and solutions in the previous stage, this stage involved translating user needs into an initial design that could be tested and evaluated. The low-fidelity (lo-fi) app prototype from the first iteration was designed to visualize the flow of user interaction and ensure that every feature and flow was user-friendly. The high-fidelity (hi-fi) prototypes from the second and third iterations were used to determine how users would interact with an app featuring visual elements and interactivity. This design also tested user flow and design consistency. Figures 2-4 present examples of the lo-fi prototype design, featuring the home screen, stroke education, and stroke rehabilitation activity targets. Figures 5-7 display examples of the hi-fi prototype design with the same content.

### Demonstration, Evaluation, and Communication

In the demonstration stage, we showed interviewees the lo-fi app prototype, and they provided feedback on the design in the evaluation and communication stages. This yielded various inputs regarding the initial design features. All reviews submitted by the interviewees were validated and analyzed to determine improvement priorities. Table 2 summarizes the lo-fi prototype evaluation, which led to improvements in the hi-fi version.

**Table 2.** Summary of low-fidelity prototype evaluations.

Improved features	Improvements
Authentication	Using a registration certificate number (STR) instead of a practice license number
User profile management features	Adding data on patients’ disease history, drug allergies, and medical records Adding an STR number for medical personnel data Linking to the national data system and restricting access to authorized medical personnel
Therapy results report submission feature	Connecting to a consultation or home-visit service Sending examination results to medical personnel in video format
Gamification (pop-up quiz)	Adding a “Skip” button for questions
Activity target features	Improving concision Adding long- and short-term activity targets
Consultation and home-visit features	Adding patient medical record data to the home-visit feature For consulting services, removing rehabilitation progress trackers

The evaluation and communication stages were carried out using surveys and interviews to collect feedback on the design. Both quantitative (SUS) and qualitative methods were used. Evaluation data for SUS were collected from 146 respondents from November 1, 2025, to November 7, 2025. The respondents who filled out the SUS questionnaire included a balanced number of men and women, with 73 respondents aged 26 to 35 years (n=146, 50%). The average SUS score was 71.83, a “Good” result [26]. Based on these results, we interviewed 20 medical personnel and 5 patients with stroke from November 7, 2025, to November 9, 2025. Multimedia Appendix 6 summarizes the recommended improvements.

The final evaluation was carried out using the PSSUQ to measure app design quality based on the users’ perceptions of ease of use and information quality. The PSSUQ was distributed from November 17, 2025, to November 21, 2025, and was returned by 128 respondents, including 69 (54%) women and 59 (46%) men. Participants aged 26 to 35 years comprised the largest group with 40 (31%) respondents, followed by the 17 to 25 group with 33 (26%) respondents. Table 3 indicates that these participants found the improved app easy to implement and use.

**Table 3.** Post-Study System Usability Questionnaire results.

Category	System usefulness	Information quality	Interface quality	Overall system
Red	2.32	2.39	2.42	2.35
Lower limit	2.12	2.19	2.23	2.16
Upper limit	2.52	2.9	2.60	2.54

The app’s features were grouped into 4 categories of health service approaches, namely promotive, preventive, curative, and rehabilitative. Promotion focuses on knowledge, awareness, and user participation so that they are proactive in maintaining their health. Prevention pertains to the avoidance or early detection of health problems through routine monitoring and assessment. Curative care concerns the treatment of conditions through access to medical services. Rehabilitation focuses on restoring cognitive, emotional, and social functions through fun, therapeutic activities.

The entire list of app features mapped to each persona is listed in Multimedia Appendix 7. Promotion is supported by onboarding, video and text education features, and rehabilitation progress monitoring. Prevention is facilitated by target activity, gamification, and rehabilitation schedule reminder features. Curative care is supported by consultation, therapy results reports, and home-visit service features. Rehabilitation is facilitated by a rehabilitation training program and user profile management to tailor rehabilitation interventions to the individual circumstances of each patient. In addition, we implemented authentication features that allow each user to securely log in and access the system with a verified identity, thereby ensuring the security and personalization of patients’ rehabilitation data.

All features were mapped onto intervention theory principles to ensure that the features had a strong interventional basis. Intervention theory focuses on 3 main aspects: valid and useful information; free, informed choices; and internal commitment [20]. Valid and useful information requires that the data provided by the system or intervention must be accurate, trustworthy, and relevant to the needs of the user [20]. Free, informed choice means that users have the liberty to make decisions without coercion or manipulation [20]. Internal commitment mandates that users have the internal motivation and commitment to carry out and complete intervention programs without any external pressure or coercion [20]. Feature mapping was carried out to document the theoretical basis of each feature, ensure the completeness of behavioral change components, and provide theoretical justifications for design decisions. Mapping each feature onto the aforementioned principles (Table 4) allowed us to evaluate how well each feature contributed to an effective intervention process.

**Table 4.** Feature mapping with intervention theory.

Persona, principle of intervention theory, and feature	Theoretical justification	
Patients who experienced a stroke		
Valid and useful information		
Onboarding	Provides accurate initial information on the app’s functionality and a thorough introduction to the app’s features prior to direct use by patients who have experienced a stroke.	
General educational video features related to stroke and stroke rehabilitation	Provide education in video format about stroke and stroke rehabilitation that is relevant to patients performing stroke rehabilitation.	
General educational articles	Provide education in article format about stroke and stroke rehabilitation that is relevant to patients performing stroke rehabilitation.	
Rehabilitation progress monitoring	Provides detailed information related to the patient’s rehabilitation progress based on the condition and the latest assessment of medical personnel.	
Rehabilitation training program	Provides education in the form of articles, videos, and short videos on stroke rehabilitation exercises at home.	
Free, informed choice		
Pop-up quiz (gamification)	Provides freedom for patients who have experienced a stroke to fill out pop-up quizzes or skip them.	
Reminders	Provide freedom for patients who have experienced a stroke to manage schedules, whether exercise schedules, consultations, or home visits.	
User profile management	Provides freedom for patients who have experienced a stroke to manage their personal data.	
Therapy reports	Provide freedom for patients who have experienced a stroke to submit therapy results to medical personnel in video format.	
Health consultation	Provides freedom for patients who have experienced a stroke to consult with and choose available medical personnel.	
Home-visit service	Provides freedom for patients who have experienced a stroke to conduct home visits and choose available medical personnel.	
Internal commitment		
Activity targets	Giving patients control over setting personal goals creates a sense of ownership, so that the motivation to achieve them comes from their own intrinsic desires, not external compulsions.	
Medical personnel		
Valid and useful information		
Onboarding	Provides accurate initial information on the app’s functionality and a thorough introduction to the app’s features before being used directly by medical personnel.	
General educational video	Allows medical personnel to manage education in video format about stroke and stroke rehabilitation by ensuring that the information provided to patients remains accurate and relevant.	
General educational articles	Allow medical personnel to manage education in article format on stroke and stroke rehabilitation by ensuring that the information provided to patients remains accurate and relevant.	
Rehabilitation progress monitoring	Allows medical personnel to update patient rehabilitation progress data validly and accurately according to the patient’s current condition.	
Rehabilitation training program	Allows medical personnel to manage education in the form of articles, videos, and short videos on home stroke rehabilitation exercises by ensuring that the information provided to patients remains accurate and relevant.	
Pop-up quiz (gamification)	Allows medical personnel to add pop-up quiz questions with correct answers.	
Therapy results report	Allows medical personnel to provide verified and accurate evaluation results for rehabilitation progress.	
Medical consultation	Allows medical personnel to serve patients who have experienced a stroke and provide accurate and verified evaluation results.	
Home-visit service	Allows medical personnel to serve patients who have experienced a stroke and provide accurate and verified evaluation results.	
Free, informed choice		
User profile management	Provides freedom for medical personnel to manage their personal data in accordance with the decisions of medical personnel.	
Internal commitment		
Activity target	Allows medical personnel to provide recommendations for targeted activities tailored to the patient’s condition. This encourages patients to have a personal stake in completing predetermined rehabilitation activities.	

## Discussion

### Principal Findings

Across 3 iterations involving doctors, therapists, medical students, and individuals interested in stroke rehabilitation issues, this study collected feedback to build an app based on patients’ and related stakeholders’ feedback. The app’s main features are grouped into several interface categories, including exercise and progress monitoring programs, consultation and home-visit services, and educational content designed to support poststroke rehabilitation. These features were mapped onto the principles of intervention theory, namely valid information; free, informed choice; and internal commitment. The evaluations of the app design suggested an increase in usage and user satisfaction, as well as success in addressing the challenges and limitations identified in previous research.

In addition, this study discovered a new problem: decreased patient ability to undergo rehabilitation in hospitals due to age. This problem was overcome by adding a feature for home visits, which remains rarely addressed in stroke rehabilitation apps [17,18,27]. Another new problem in the Indonesian context concerns the mental struggles of patients, which impede rehabilitation. For example, low patient motivation to carry out stroke rehabilitation was overcome by adding activity targets to the app, thus ensuring consistency and triggering patient motivation.

In Indonesia, there is a lack of objective evidence regarding home care because evaluations rely on subjective patient reports. This problem was addressed by designing a rehabilitation progress monitoring feature and therapy result reports, integrated with patient medical records in the app; this allows patient progress to be evaluated properly, rather than relying solely on oral testimonials. Thus, several features of the app were adapted to the context in Indonesia, including home-visit features, rehabilitation training programs, gamification (pop-up quizzes), rehabilitation progress monitoring, activity targets, and therapy reports.

### Theoretical Implications

Researchers have previously conducted systematic reviews or usability assessments of existing systems using various evaluation frameworks [15,17,18,27,28]. In contrast, this study designed a stroke rehabilitation management app by consulting patients and medical personnel using DSR. Thus, the feedback obtained represents the true needs of patients who experienced a stroke, including recovery processes and mechanisms underlying behavioral changes, as well as the promotive, preventive, curative, and rehabilitative aspects of health promotion. This study also expands the limited literature on stroke rehabilitation in developing countries [11].

There is a gap in the research [15,17] regarding the low adherence of users to rehabilitation procedures, which we addressed by adding activity target features and schedule reminders. This will help patients, families, and caregivers monitor patient rehabilitation in a more structured and scheduled manner. Another gap [18] is the limited variety of content for different stages of stroke and patient conditions, which we addressed by providing a range of educational content and exercises to suit individual needs. The app also provides tutorials on usage and onboarding, which were not included in the research conducted by Song et al [18]. Furthermore, previous research used relatively small samples and did not include all relevant respondents, especially patients [28]. Our app also minimizes or eliminates certain challenges of stroke rehabilitation. The app overcomes the limitations of health facilities by providing consultation and home-visit services and increasing digital literacy and compliance with rehabilitation exercises. The app can also mitigate the impact of stroke on patients by optimizing the body’s functionality and preventing complications.

The importance of this study stems from the high prevalence of stroke in Indonesia and the lack of applications that support patient-centered stroke rehabilitation. Research in Canada [17], Belgium [15], and the United States [18] has discussed various mHealth apps for stroke rehabilitation, covering improved physical function, physical activity, and patient quality of life. However, these are all developed countries, and no previous work has focused on Indonesian patients with stroke. The problems identified in our interviews reflect those noted in previous works [15,17]. Furthermore, the app offers solutions such as schedule reminder features, gamification in the form of pop-up quizzes, and rehabilitation progress monitoring.

### Practical Implications

For health care facilities, such as hospitals, therapy clinics, and health centers, our app can be used to monitor patients undergoing stroke rehabilitation or to help prevent recurrent strokes. For medical personnel, the app can help carry out rehabilitation services both online (through messages or voice media) and offline (by visiting the patient’s home). This would make rehabilitation services more efficient and help evaluate the rehabilitation process based on patient medical data. Medical personnel can also provide education and training on stroke rehabilitation at home. Zeng et al [29] noted that improved health literacy among stroke survivors and caregivers improves self-management for survivors.

For patients, families, and caregivers, the app can provide educational information about stroke rehabilitation, including exercises that can be performed at home easily and affordably. Stroke apps should involve online communities to support survivors and caregivers [16,30]. Moreover, the app facilitates consultation services and home visits from medical personnel. There are also schedule reminders, activity targets, and progress-tracking features for patients, families, and caregivers to monitor speech, occupation, and function.

For mHealth developers, this app is built to serve the needs and expectations of users in Indonesia. Likewise, for the government or policymakers, it can be connected to hospitals and official services. The app can be used to collect data on patient activity patterns after stroke and to provide evidence of its effectiveness in supporting stroke rehabilitation. The government should consider the value of medical personnel being able to access educational and training information for patients, families, and caregivers.

### Limitations

Despite these strengths, the study has limitations, such as limited digital literacy among participants, which made it difficult for them to understand certain features or use the app independently. Therefore, training on using the system is needed. Other difficulties included technical limitations when using the application, such as internet connection problems, device availability, operating system compatibility, and device storage capacity.

Digital apps can be difficult for older adults to use. Most of our participants were young adult caregivers of patients with stroke, which explains the lack of older participants. It was difficult to accommodate all users due to the limited accessibility of technology in remote areas and the age of patients who experienced a stroke. Although older patients may have difficulty using the app independently, caregivers, families, or online communities can help them use features that present challenges, ensuring that the collected data represent the needs of all age groups.

Further research is recommended to conduct longitudinal analyses measuring long-term adherence, app usage sustainability, and the impact on rehabilitation outcomes among patients who have experienced a stroke. Researchers could also adopt supportive technologies, such as wearable devices, to make the app accessible to users with limitations.

## Supplementary material

10.2196/91464 Multimedia Appendix 1 System Usability Scale (SUS) questionnaire questions.

10.2196/91464 Multimedia Appendix 2 Post-Study System Usability Questionnaire (PSSUQ) questionnaire questions.

10.2196/91464 Multimedia Appendix 3 Interview questions.

10.2196/91464 Multimedia Appendix 4 Summary of problems.

10.2196/91464 Multimedia Appendix 5 Problem solution mapping with the “how might we” technique.

10.2196/91464 Multimedia Appendix 6 Summary of high-fidelity prototype improvements.

10.2196/91464 Multimedia Appendix 7 Feature summaries.

## Abbreviations

DSR: design science research
hi-fi: high-fidelity
HMW: how might we
lo-fi: low-fidelity
mHealth: mobile health
PSSUQ: Post-Study System Usability Questionnaire
SUS: System Usability Scale
WHO: World Health Organization
