# Supplementary material for: Designing an mHealth App for Stroke Rehabilitation in Indonesia: Mixed Methods Design Science Research Study
Source: JMIR Rehabil Assist Technol. 2026 Jul 23;13:e91464. doi: 10.2196/91464 (PMC13394849; doi:10.2196/91464)
Supplement: Multimedia Appendix 1 [file rehab-v13-e91464-s001.docx]

## Multimedia Appendix 1: SUS Questionnaire Questions

| Code | Research Questions |
| --- | --- |
| S1 | I think I will use this app a lot. |
| S2 | In my opinion, this app is not too complicated. |
| S3 | I find this app to be easy to use. |
| S4 | I feel like I need help to be able to use this app . |
| S5 | I feel like the various functions in this app are well integrated. |
| S6 | In my opinion, there are too many inconsistencies in this app. |
| S7 | I imagine that most people will learn to use this app very quickly. |
| S8 | I find this app very complicated/awkward to use. |
| S9 | I feel very confident using this app. |
| S10 | I need to learn a lot before I can use this app. |
