# Supplementary material for: Designing an mHealth App for Stroke Rehabilitation in Indonesia: Mixed Methods Design Science Research Study
Source: JMIR Rehabil Assist Technol. 2026 Jul 23;13:e91464. doi: 10.2196/91464 (PMC13394849; doi:10.2196/91464)
Supplement: Multimedia Appendix 2 [file rehab-v13-e91464-s002.docx]

## Multimedia Appendix 2: PSSUQ Questionnaire Questions

| Dimension | Code | Research Questions |
| --- | --- | --- |
| System Usefulness (SYSUSE) | Q1 | Overall, I am satisfied with the ease of use of this app. |
|  | Q2 | The app is easy to use. |
|  | Q3 | I can quickly complete tasks and scenarios using this app. |
|  | Q4 | I feel comfortable using this app. |
|  | Q5 | I easily learned how to use this app. |
|  | Q6 | I believe I can be productive quickly using this app. |
| Information Quality (INFOQUAL) | Q7 | The system gave me an error message telling me how to fix the problem. |
|  | Q8 | When I make a mistake in using this app, I can recover it easily and quickly. |
|  | Q9 | Information (help, messages, and other documentation) is provided in the app clearly. |
|  | Q10 | The information I needed was easy to find. |
|  | Q11 | This information was effective in helping me complete tasks and scenarios. |
|  | Q12 | The mapping of information on the application is clear. |
| Interface Quality (INTERQUAL) | Q13 | The app's interface is fun. |
|  | Q14 | I love using the interface of this app. |
|  | Q15 | This app has all the functions and capabilities I expected. |
|  | Q16 | Overall, I am satisfied with this app. |
