# Supplementary material for: Designing an mHealth App for Stroke Rehabilitation in Indonesia: Mixed Methods Design Science Research Study
Source: JMIR Rehabil Assist Technol. 2026 Jul 23;13:e91464. doi: 10.2196/91464 (PMC13394849; doi:10.2196/91464)
Supplement: Multimedia Appendix 3 [file rehab-v13-e91464-s003.docx]

## Multimedia Appendix 3: Interview Questions

| Purpose | Questions |
| --- | --- |
| General insights and personal experience | As far as you know, how is the stroke rehabilitation process? Is it ideal? If not, what would be the ideal? |
|  | What do you think are the characteristics of stroke patients who need rehabilitation? |
|  | What types of rehabilitation are generally given to stroke patients? |
|  | Who is ideally involved in the rehabilitation process of stroke patients? |
|  | How often do stroke patients need to undergo rehabilitation sessions in one week? |
|  | How is the process of referring stroke patients to rehabilitation services done in your place? |
|  | Which healthcare facilities do you know that provide stroke rehabilitation services? |
| Needs and challenges in stroke rehabilitation | What do you think are the challenges in stroke rehabilitation? |
|  | Are the sources of information and methods currently available sufficient to address the challenges in stroke rehabilitation? |
|  | What skills and knowledge do you think are needed to effectively manage stroke patients at different levels of stroke? |
|  | How important do you think your involvement and attitude as a doctor/therapist are in the rehabilitation process of stroke patients? |
| Perception of stroke rehabilitation applications | Have you used a health app before? If so, what is your impression of the app? |
|  | What is your view on the future usefulness of mobile apps for stroke rehabilitation management? |
|  | What is the role of the app in the context of stroke patients with physical or cognitive limitations? |
|  | Do you feel that stroke patients or their families have enough digital literacy to use stroke rehabilitation apps independently? |
|  | How does the use of digital tools or mobile apps affect you in caring for patients, especially stroke patients? |
|  | How do you view the benefits of mobile/telehealth apps in your daily practice for stroke rehabilitation? |
|  | Do you think there is a difference in the need for features between the early and advanced phases of stroke rehabilitation? |
| Issues, concerns, and app evaluation | Do you see any problems, drawbacks, or shortcomings of the existing mobile app-based rehabilitation programs? |
|  | Are there any obstacles you face while using the stroke rehabilitation application? |
|  | What are some of the biggest needs and concerns regarding the adoption of technologies such as applications for stroke rehabilitation management? |
| Ideal feature and design requirements | What features do you feel you need to have in a stroke rehabilitation application? Why? |
|  | In your opinion, how ideal is the process of evaluating the progress of stroke patients through the application? |
|  | Do you think stroke rehabilitation management apps should focus on patients, families, or medical personnel? Or a combination of all three? Why? |
|  | How do you think the app can better support communication and planning for medical personnel with patients? |
| Recommendations and suggestions | What are your suggestions for the future improvement of this mobile app-based stroke rehabilitation program? |
|  | If there is a stroke rehabilitation management app, would you recommend it to your relatives, friends, or neighbors? Why? |
| Challenges in stroke rehabilitation | What do you find most difficult in the first weeks after a stroke? |
|  | Are you experiencing physical impairment as a result of a stroke? If so, what are they? |
|  | Do you have difficulty speaking or understanding the language? |
|  | Are you currently or have you previously undergone stroke rehabilitation? If so, where did you undergo stroke rehabilitation? |
|  | Do you feel that progress is being made when your rehabilitation is going slowly, quickly, or as expected? Why? |
|  | What are the challenges or obstacles you face while undergoing stroke rehabilitation? |
|  | Have you ever wanted to give up or feel hopeless in the process of rehabilitation? What causes these feelings? |
| Needs in stroke rehabilitation | What do you need in stroke rehabilitation? |
|  | Do you feel stressed, anxious, or depressed after having a stroke? |
|  | Have you ever received assistance from a psychologist, counselor, or stroke patient support group? |
|  | What kind of support do you need most right now to stay motivated to go through rehabilitation? |
|  | Do you get enough information about stroke and the rehabilitation process from your doctor, therapist, or other sources? |
|  | What kind of information do you think is most important but haven't gotten about stroke rehabilitation? |
|  | What is the role of family or close people in accompanying you during stroke recovery? |
|  | If you could suggest a change or improvement in stroke rehabilitation services, what would you like to change? |
| Rehabilitation process | What was the rehabilitation process during your hospitalization? And after being discharged from the hospital? |
| Initial experience with stroke rehabilitation apps | Previously, did you have any experience in using a mobile application-based rehabilitation program? What was your experience in using the app? |
|  | Have you heard of or used apps that can help with the stroke rehabilitation process before? |
| Overview and benefits of the app | What is your view on the future usefulness of mobile apps for stroke rehabilitation management? |
|  | How do you view the benefits of mobile/telehealth apps for stroke rehabilitation? |
|  | Do you think this app helps you get better at your daily activities? |
| Needs, features, and support | What features do you need in stroke rehabilitation? Why? |
|  | What are some of the biggest needs and concerns regarding the adoption of technologies such as applications for stroke rehabilitation management? |
|  | How do you feel that this mobile app-based rehabilitation program supports your rehabilitation practice? |
| Evaluation of existing applications | Do you see any problems, drawbacks, or shortcomings of the existing mobile app-based rehabilitation programs? |
| Motivation and support role | What motivates you to continue doing rehabilitation exercises? |
|  | How important is your family's role in helping you undergo exercise or treatment after a stroke? |
|  | What do you think is the role of healthcare workers' involvement and attitudes in your rehabilitation process? What are some aspects that make a healthcare worker relevant to you? |
| Recommendations and suggestions | If there is a stroke rehabilitation management app, would you recommend it to your relatives, friends, or neighbors? Why? |
|  | What are your suggestions for the future improvement of this mobile app-based stroke rehabilitation program? |
