# Supplementary material for: Designing an mHealth App for Stroke Rehabilitation in Indonesia: Mixed Methods Design Science Research Study
Source: JMIR Rehabil Assist Technol. 2026 Jul 23;13:e91464. doi: 10.2196/91464 (PMC13394849; doi:10.2196/91464)
Supplement: Multimedia Appendix 4 [file rehab-v13-e91464-s004.docx]

## Multimedia Appendix 4: Summary of Problems

| **Problems** | **Sample Quotes** | **Number of Interviewees** | **Percentages of Interviewees (n=18)** |
| --- | --- | --- | --- |
| A variety of conditions between post-stroke patients, such as variations in functional abilities, speech, and independence. | "*The mental condition of patients who are not supportive in rehabilitation such as rejection of illness, lack of family support, laziness, and others. This condition can affect the outcome of stroke rehabilitation so that it will be more difficult for patients to recover and carry out their activities as before."* (Respondent 1, Female). | 12 | 66.67% |
| Lack of self-rehabilitation exercises at home | *"The lack of home exercises recommended by therapists or medical personnel is also a challenge in stroke rehabilitation. This usually happens because evaluations for home rehabilitation are only asked during rehabilitation sessions without any evidence of training."* (Respondent 1, Female). | 11 | 61.11% |
| Low adherence to patient training | *"Low patient practice compliance, uneven distribution of health services, and undertrained rehabilitation personnel."* (Respondent 7, Female). | 9 | 50.00% |
| Lack of role and willingness of medical personnel | *"The lack of medical rehabilitation doctors and health service facilities in the area where the resource persons are located, the cost of rehabilitation, patients rarely or do not do independent exercises"* (Respondent 10, Female). | 9 | 50.00% |
| There is no evidence of home exercises as a form of evaluation, so evaluation only relies on patient reports which are often subjective and not always accurate. | "*. Another challenge also comes when patients rarely do self-rehabilitation and there is no tracking evaluation of the rehabilitation results."* (Respondent 6, Male). | 8 | 44.44% |
| Decreased ability of patients to undergo rehabilitation in the hospital due to age | "*The condition of the patient is elderly, so it is difficult to do rehabilitation exercises."* (Respondent 12, Male). | 7 | 38.89% |
| Limited health service facilities in carrying out the rehabilitation process | *"Not all health centers have multidisciplinary teams (doctors, nurses, therapists, psychologists, and others), lack of access to health services in remote areas, inadequate technology, and lack of emotional support."* (Respondent 6, Male). | 6 | 33.33% |
