# Supplementary material for: Designing an mHealth App for Stroke Rehabilitation in Indonesia: Mixed Methods Design Science Research Study
Source: JMIR Rehabil Assist Technol. 2026 Jul 23;13:e91464. doi: 10.2196/91464 (PMC13394849; doi:10.2196/91464)
Supplement: Multimedia Appendix 5 [file rehab-v13-e91464-s005.docx]

Multimedia Appendix 5: Problem solution mapping with the “how might we” technique

| **Problem group** | **HMW question** | **Interviewee group** | **Proposed feature** | **Feature explanation** |
| --- | --- | --- | --- | --- |
| Lack of self-rehabilitation exercises at home | How can we help patients be more diligent in performing rehabilitation exercises at home? | Patients | Features of the rehabilitation training program | This feature provides step-by-step guidance on different types of rehabilitation, providing with a variety of instructions, visuals, and audio. This feature helps patients’ complete rehabilitation pro at home. |
|  |  |  | Features of general educational texts related to stroke and stroke rehabilitation | This feature provides a variety of educational content in the form of articles about stroke rehabilitation. |
|  |  |  | General educational videos related to stroke and stroke rehabilitation | This feature provides educational content in the form of videos about stroke and stroke rehabilitation. |
| The patient’s mental condition is not conducive to rehabilitation; for instance, a lack of motivation or enthusiasm | How can we help patients overcome mental barriers so they can undergo rehabilitation properly? | Patients | Activity targets | This feature assigns and tracks short- and long-term rehabilitation goals to motivate patients. |
| Low patient adherence to training | How can we improve patient adherence to rehabilitation exercise schedules? | Patients, medical personnel | Schedule reminders | This feature provides automatic notifications that remind patients of exercise times, medication consumption, or appointments with medical personnel, which can be customized according to the patient’s daily routine. |
|  |  |  | Gamification: pop-up quizzes | This feature provides questions that can be answered by users of the rehabilitation training program. |
|  |  |  | Rehabilitation progress monitoring | This feature tracks the patient’s progress in various aspects of rehabilitation, including progress graphs, daily/weekly exercise statistics, and target achievement. |
| There is no evidence on home exercises as a form of evaluation, so evaluation relies solely on patient reports, which are often subjective and not always accurate | How can we ensure that there are evidence or records of a patient’s rehabilitation practice at home? |  |  |  |
|  |  |  | Therapy results reports | This feature sends progress reports and exercise data to medical teams that treat patients to improve coordination between medical personnel. |
| Lack of clear roles and willingness among medical personnel | How can more medical personnel play a role in the patient rehabilitation process? | Patients, medical personnel | Consulting services | This feature allows patient consultations with doctors, physiotherapists, or other medical personnel via chat, video call, or voice call. |
| Limited healthcare facilities |  |  |  |  |
| Decreased ability of patients to undergo rehabilitation in the hospital due to age | How can the rehabilitation program be adapted to patients’ decreasing capacities with age? | Patients, medical personnel | Home visit services | This feature books medical personnel visits to patients’ homes; includes scheduling, selection of rehabilitation service types, cost information, and tracking of visit status. This feature targets patients with limited mobility. |
| A wide range of conditions among post-stroke patients | How can we make it easier for patients to convey their conditions or complaints? | Patients | User profile management | This feature records the data and conditions of patients and medical personnel through complete inputs that store demographic information, medical history, and conditions; thereby, other features, such as education features or activity monitoring, can be tailored to the patient’s condition. |
