# Supplementary material for: Designing an mHealth App for Stroke Rehabilitation in Indonesia: Mixed Methods Design Science Research Study
Source: JMIR Rehabil Assist Technol. 2026 Jul 23;13:e91464. doi: 10.2196/91464 (PMC13394849; doi:10.2196/91464)
Supplement: Multimedia Appendix 6 [file rehab-v13-e91464-s006.docx]

## Multimedia Appendix 6. Summary of High-fidelity Prototype Improvement Suggestions Referring to SUS Results and Interviews

| **Features** | **Suggestions** | **Improvements** |
| --- | --- | --- |
| Therapy results report | The progress video feature is considered ineffective and actually burdens patients. | Making the therapy results report feature optional for patients to do after using consultation services or home visits. |
| All features | The font size is considered too small. | Increase the font size for each page in the app. |
| Not associated with any features | There is no feature to adjust the font size according to the needs and desires of the user. | Provides settings for the selection of text sizes consisting of three categories, including small, medium, and large, so that users can choose the text size according to the user's needs and desires. |
| Not associated with any features | The app doesn't have an onboarding or usage tutorial for new users yet. | Provide onboarding or application usage tutorials by providing an explanation of each feature for new users. |
| Rehabilitation Progress Monitoring | The motor assessment method used is not suitable for stroke patients. | Converting motor assessment to functional assessment using the Motor Assessment Scale (MAS) method with the aim of complying with functional monitoring for stroke patients |
| All features | The color scheme of the display is considered monotonous and less attractive. | Add color gradations and other colors to make them look more attractive. |
