# Supplementary material for: Designing an mHealth App for Stroke Rehabilitation in Indonesia: Mixed Methods Design Science Research Study
Source: JMIR Rehabil Assist Technol. 2026 Jul 23;13:e91464. doi: 10.2196/91464 (PMC13394849; doi:10.2196/91464)
Supplement: Multimedia Appendix 7 [file rehab-v13-e91464-s007.docx]

## Multimedia Appendix 7. Features Summaries

| **Persona** | **Features** | **Feature Description** | **Implemented Health Service** |
| --- | --- | --- | --- |
| Post-stroke patients | Onboarding | The post-stroke patient onboarding feature is a feature that provides an explanation and illustration of the main functions or features of the application to help new users understand the use of each feature before actually using the application. | Promotive, because it helps patients to understand the functionality of the application and the overall features of the application before using the application independently. |
|  | User Profile Management | The post-stroke patient user profile management feature is a feature that provides post-stroke patient personal data that can be managed according to the patient's wishes. | Rehabilitative, as it helps patients to **personalize rehabilitation interventions** tailored to the specific conditions of post-stroke patients in their profile. |
|  | Rehabilitation Progress Monitoring | The post-stroke patient rehabilitation progress monitoring feature is a feature that provides all details of the patient's current rehabilitation progress condition, such as functional progress, speech progress, and occupancy progress. | Promotive, because it helps patients to inform the progress of rehabilitation and the patient's current condition. |
|  | Schedule Reminder | The post-stroke patient schedule reminder feature is a feature that provides all data on the patient's exercise schedule, consultation schedule, and home visit schedule that can be managed by him. | Preventive, because it helps patients to manage all schedules of exercises, consultations, and home visits regularly to prevent worsening of the patient's condition. |
|  | Activity Target | The post-stroke patient activity target feature is a feature that provides all activity data that needs to be done by the patient with two main categories, the activity target recommended by the medical personnel and the personal activity target that can be managed by him. | Preventive, because it helps patients to manage all target activities that need to be done by the patient to prevent worsening of the patient's condition or stroke recurrence. |
|  | Rehabilitation Training Program | The rehabilitation training program feature is a feature that can be seen by patients in the form of articles, videos, or short videos containing procedures for stroke rehabilitation exercises at home. | Rehabilitative, because it helps patients to undergo rehabilitation through video content or articles. It corresponds to rehabilitative that focuses on accurate and structured cognitive training. ( AbdulRaheem , 2023) |
|  | Gamification (Quiz) | The gamification feature of pop up quiz is a feature that is provided in the form of pop up questions and can be skipped or filled in by patients as a form of entertainment and increases the patient's activeness in using the application. | Preventive, because it helps patients to maintain and increase motivation in seeing rehabilitation exercise programs. |
|  | Consulting Services | The post-stroke patient consultation service feature is a feature that provides consultation services with available medical personnel and can provide service results in the form of a resume that contains descriptions of results, suggestions, recommendations for exercise programs, and recommendations for targeted activities that need to be considered and carried out by post-stroke patients. | Curative, because it helps patients to recover from their condition or illness through consultation services and get evaluation results, recommendations for activity targets, and recommendations for rehabilitation training programs. |
|  | Therapy Results Report | The therapy results report feature is a feature provided to send rehabilitation results after a few days of consulting services or home visits to see the progress of post-stroke patient rehabilitation and monitor the follow-up exercise program that needs to be carried out. | Curative, because it helps patients to provide rehabilitation results through video and obtain evaluations, recommendations for activity targets, recommendations for rehabilitation training programs, and results of rehabilitation progress assessments from medical personnel. |
|  | Home Visit Service | The post-stroke patient home visit service feature is a feature that provides home visit services with medical personnel who will come to the home and provides service results in the form of a resume that contains a description of results, suggestions, exercise program recommendations, activity target recommendations, and rehabilitation progress assessments that need to be considered by post-stroke patients. | Curative, because it helps patients to recover from their condition or illness through home visit services by bringing medical personnel directly to the home and providing evaluations, recommendations for activity targets, and recommendations for rehabilitation training programs. |
|  | Educational Video related to Stroke and Stroke Rehabilitation | The general education video feature related to stroke is a feature that can be viewed by patients in the form of videos or short videos containing general education related to stroke and stroke rehabilitation. | Promotive, because it helps patients to get general information related to stroke and stroke rehabilitation in video and short video formats. |
|  | Educational Texts related to Stroke and Stroke Rehabilitation | The feature of general education articles related to stroke is a feature that can be seen by patients in the form of articles containing general education related to stroke and stroke rehabilitation. | Promotive, because it helps patients to get general information related to stroke and stroke rehabilitation in article or text format. |
| Medical Personnel | Onboarding Features | The medical onboarding feature is a feature that provides an explanation and illustration of the main functions or features of the application to help new users understand the use of each feature before actually using the application. | Promotive, because it helps medical personnel to understand and recognize the functionality of the application, including the introduction of all the features of the application. |
|  | User Profile Management | The user profile management feature for medical personnel is a feature that provides personal data of medical personnel that can be managed as they wish. | Rehabilitative, because it helps medical personnel to **personalize data so that patients can choose medical personnel**  who are able to handle their condition. |
|  | Rehabilitation Training Program | The medical personnel rehabilitation exercise program feature is a feature that provides the entire content of the exercise program that can be managed by medical personnel to provide home exercise program assistance to post-stroke patients. | Rehabilitative, because it helps medical personnel to provide training educational content to patients and help with patient rehabilitation. |
|  | Gamification | The gamification feature of the medical personnel pop up quiz is a feature that provides input of question data and can be managed directly by medical personnel. | Preventive, because it helps medical personnel to provide questions to maintain and increase patient motivation in seeing rehabilitation exercise programs. |
|  | Consulting Services | The medical personnel consultation service feature is a feature that provides all patient data who wants to consult, consult with patients, and provide data input for the results of consultation services. | Curative, because it helps medical personnel to provide consultation services and provide evaluation results, recommendations for activity targets, and recommendations for rehabilitation training programs to prevent the deterioration of the patient's condition or the occurrence of recurrent strokes. |
|  | Home Visit Service | The home visit service feature for medical personnel is a feature that provides all data on patients who want to make home visits, conduct home visits with patients, and provide data input for the results of home visit consultation services. | Curative, because it helps medical personnel to provide home visit services and provide evaluation results, recommendations for activity targets, recommendations for rehabilitation training programs, and assessment of rehabilitation progress to prevent the deterioration of the patient's condition or the occurrence of recurrent strokes. |
|  | Activity Target | The activity target feature is a feature that is provided to add activity targets to the patient and according to their condition. | Preventive, because it helps medical personnel to provide activities that can be done by patients regularly to prevent worsening of the patient's condition. |
|  | Rehabilitation Progress Monitoring | The medical personnel rehabilitation progress monitoring feature is a feature provided to add data on patient rehabilitation progress ranging from functional progress, speech progress, and occupation progress. | Curative, because it helps medical personnel to provide an assessment of rehabilitation progress to monitor the patient's condition and prevent the deterioration of the patient's condition or the occurrence of recurrent strokes. |
|  | Educational Video Features related to Stroke and Stroke Rehabilitation | The general education video feature related to stroke and stroke rehabilitation for medical personnel is a feature that provides all general educational content related to stroke and stroke rehabilitation in the form of short videos and videos that can be managed by medical personnel to provide general understanding assistance to post-stroke patients. | Promotive, because it helps medical personnel to manage general information related to stroke and stroke rehabilitation in video and short video formats. |
|  | Educational Texts related to Stroke and Stroke Rehabilitation | The general educational text feature related to stroke and stroke rehabilitation for medical personnel is a feature that provides all general educational content related to stroke and stroke rehabilitation in the form of articles that can be managed by medical personnel to provide general understanding assistance to post-stroke patients. | Promotive, because it helps medical personnel to manage general information related to stroke and stroke rehabilitation in text or article format. |
|  | Therapy Results Report | The medical personnel therapy results report feature is a feature that provides all patient progress data in the form of videos and provides data input for evaluation results to post-stroke patients. | Curative, because it helps medical personnel to see the progress of the patient's rehabilitation through video and provide evaluation results, recommendations for activity targets, and recommendations for rehabilitation training programs to prevent the patient's condition from deteriorating or the occurrence of recurrent strokes. |
